# Optimisation of Sensor and Sensor Node Positions for Shape Sensing with a Wireless Sensor Network—A Case Study Using the Modal Method and a Physics-Informed Neural Network

**DOI:** 10.3390/s25175573

**Published:** 2025-09-06

**Authors:** Sören Meyer zu Westerhausen, Imed Hichri, Kevin Herrmann, Roland Lachmayer

**Affiliations:** Institute of Product Development, Leibniz University Hannover, An der Universität 1, 30823 Garbsen, Germany; imed.hichri@stud.uni-hannover.de (I.H.); herrmann@ipeg.uni-hannover.de (K.H.); lachmayer@ipeg.uni-hannover.de (R.L.)

**Keywords:** structural health monitoring, shape sensing, optimal sensor placement, wireless sensor network, sensor node position optimisation

## Abstract

Data of operational conditions of structural components, acquired, e.g., in structural health monitoring (SHM), is of great interest to optimise products from one generation to the next, for example, by adapting them to occurring operational loads. To acquire data for this purpose in the desired quality, an optimal sensor placement for so-called shape and load sensing is required. In the case of large-scale structural components, wireless sensor networks (WSN) could be used to process and transmit the acquired data for real-time monitoring, which furthermore requires an optimisation of sensor node positions. Since most publications focus only on the optimal sensor placement or the optimisation of sensor node positions, a methodology for both is implemented in a Python tool, and an optimised WSN is realised on a demonstration part, loaded at a test bench. For this purpose, the modal method is applied for shape sensing as well as a physics-informed neural network for solving inverse problems in shape sensing (iPINN). The WSN is realised with strain gauges, HX711 analogue-digital (A/D) converters, and Arduino Nano 33 IoT microprocessors for data submission to a server, which allows real-time visualisation and data processing on a Python Flask server. The results demonstrate the applicability of the presented methodology and its implementation in the Python tool for achieving high-accuracy shape sensing with WSNs.

## 1. Introduction

The integration of sensors into structural components is facing an increasing interest, especially in the field of structural health monitoring (SHM) in aerospace applications [1]. Such integrated sensors allow, for example, the detection of damage and the monitoring of damage growth. This is especially of interest since aerospace components have to be damage-tolerant until a certain level, and therefore, the detection and characterisation of damage is crucial. A research field with increasing interest in the field of SHM is the so-called shape and load sensing. This field utilises sensor measurements at discrete positions, for example, with strain gauges or fibre optic sensors (FOS), to reconstruct the deformation and load field of a whole structural component [2]. This information is of great interest from a product development point of view, as it allows for the monitoring of the part for classical SHM purposes and also enables the building of knowledge on structural behaviour to adapt and optimise components for the next product generation [3,4]. Since this information becomes an integral part of these components themselves, they are called “gentelligent” in the paradigm of technical inheritance to underline the characteristic of product generation-based adaptions [5]. However, the accuracy and reliability of this acquired data are crucial for making well-informed decisions regarding design optimisations. This makes it necessary to perform an optimal sensor placement (OSP) before the sensors are integrated into the product and cannot be repositioned without damage [6]. Such integrated or embedded sensors in SHM applications could be found in various use cases in the literature. For example, Mieloszyk et al. applied embedded fibre Bragg gratings (FBG) to monitor loads of composite marine structures [7]. Zhao et al. used 3D-printed strain gauges for load monitoring with the goal of application in space components [8]. This use was also demonstrated by Haentzsche et al. on the example of a scaled wind turbine blade, where static loads were monitored in an SHM application with composite embedded strain sensors [9]. However, for large-scale components and area-wide monitoring, the use of a wireless sensor network (WSN) might be sensible. Such WSNs were, for example, applied to the example of SHM of wind turbine blades as well [10,11]. These WSNs consist of one or more sensor nodes, where one or more sensors are connected to a microcontroller that transmits their measurements wirelessly to a collection point. A problem in that matter is, however, to choose the correct positions for the sensors, but also for the microprocessors, to ensure minimal cable length between sensors and microprocessors to reduce weight from cables and possible influences on the signal quality during cable-based transmissions [12]. This optimisation for both the sensors and the microcontrollers is often not considered in a co-design approach in the literature. One study addressing this issue is found in [13], which only proposed a methodology on that issue without realisation and verification. However, it is promising to consider optimising sensor and microprocessor positions to maximise field information reconstruction accuracy by considering the influences from both positions.

Therefore, a study is presented in the following, where the methodology proposed in [13] is applied on a real-world structure on a test bench, where applied loads and resulting deformation fields should be monitored online in near-real time by using a WSN. This should demonstrate the methodology for future applications on further scaled and sensor-integrating components. For this purpose, this paper is structured as follows. After this introduction, an overview of the state of the art is presented in Section 2. Therefore, the current state of the art in the field of shape and load sensing is presented and described, with a special focus on two techniques applicable to this study, as well as known works and solutions for WSNs in that area. Afterwards, the methodology applied for the optimal sensor placement (OSP) for the sensors and the microprocessors is described in Section 3. This methodology’s implementation in Python 3.10 is then described and applied in Section 4 in a case study for the above-mentioned structure on a test bench. The results are then discussed in Section 5 regarding applicability, accuracy, and scalability. Last, Section 6 concludes this paper and gives an outlook on future works.

## 2. State of the Art

In the following, the state of the art and related works addressing the problem described above are presented. Therefore, a general description of shape sensing and available techniques is given, as well as a more detailed description of the methods further used in the application described in Section 4. Furthermore, since the application incorporates a WSN, an overview of WSN functionalities and applications is given in this section.

### 2.1. Shape Sensing in Structural Health Monitoring

The research field of shape sensing enables a near-real-time monitoring of displacement fields from discrete strain measurements. Besides, it has shown the potential to be enhanced to the so-called shape and load sensing, where not only the displacements but also the external loads are identified by strain measurements at discrete sensor positions [9]. However, the known shape sensing techniques in literature can be sorted into the four following groups as presented by the review of Gherlone et al. [2]:

1. Methods based on the numerical integration of experimental strains, like Ko’s displacement theory, which relies on the integration of strain measurements on beam-like structures into equations relying on the Euler-Bernoulli beam theory [14,15].
2. Methods using global or piecewise continuous basis functions to approximate the displacement field, like the modal method (MM), which utilises the connection between modal matrices for strains and displacements from the mode shapes of a part to estimate the displacement field of various forms of structures from strain measurements [16].
3. Methods employing Artificial Neural Networks (ANN), like, e.g., conventional neural networks, trained on specific load cases [17,18] or so-called physics-informed neural networks (PINNs), which integrate the laws of physics from structural mechanics into the training process [19,20].
4. Methods based on a finite-element discrete variational principle, like the inverse finite element method (iFEM), which utilises specific inverse element formulations on a discretised model to calculate the displacements from the strain values at specific elements [21,22].

Besides the techniques mentioned above, there could be further techniques found, like in the 2024 study of Pham et al., where an inverse mapping method is proposed and demonstrated for aerodynamic shape and load estimation [23]. Furthermore, hybrid methods, like, e.g., coupling Ko’s displacement theory with the MM [6,24] or iFEM with the MM [25], could be found in literature.

However, from conducted studies, for example, by Esposito et al. 2021, it is observable that especially the MM is robust when it comes to deviations from idealised material conditions between simulations and real-world applications, and it requires a realisable quantity of sensors for measurements [6,26]. Therefore, it is well-suited for further consideration in the application and is described in the following in more detail. Besides the MM, PINNs showed great potential for making very accurate deformation field reconstructions with few sensors, as, e.g., demonstrated by Go et al. 2025 on the example of a plate structure with a max. of five strain gauges [20]. Therefore, PINNs are further considered in the application as well and are described in more detail in the following, too.

#### 2.1.1. Modal Method

The modal method (MM) was initially introduced by Foss and Haugse and is based on a modal transformation algorithm [16]. So, it utilises modal characteristics of a structure for the estimation of displacements from discrete strain data. The strains and displacements could be expressed according to Equations (1) and (2), using a modal transformation from structural dynamics and an FE discretisation of the displacement field.

(1) $$u=\phi_{d}q$$

(2) $$\varepsilon =\phi_{s}q$$

In these equations, q denotes the *M* modal coordinates, $\phi_{d}$ is the displacements modal shape matrix and $\phi_{s}$ is the strain modal shape matrix, where every i-th column contains the information of the *i*-th mode shape [6]. The mode shapes could be computed in an FE analysis, and on this basis, the mode shape matrices are derived [27]. Rearranging Equation (1) and implementing it into Equation (2) yields

(3) $$u=\phi_{d}\phi_{s}^{-1}\varepsilon$$

which allows to calculate the displacements based on the modal matrices and the strains. In practical and real-world situations, the number of strains *S* is unlikely to be equal to the number of modes *M*. To allow for dealing with the resulting non-square matrices, the least square approach employing the Moore-Penrose inverse matrix allows to rewrite Equation (3) to

(4) $$u=\phi_{d}{\left(\phi_{s}^{T}\phi_{s}\right)}^{-1}\phi_{s}^{T}\varepsilon .$$

This approach to calculate the displacements for *S* > *M* [28]. To limit the number of modes, it is essential to find the modes that can represent the static deformation of a structure under the considered load case, thereby applying this condition. For this purpose, the procedure of Bogert et al. could be applied [29]. This procedure allows the calculation of the least-squares fit of the modal coordinates to the static solution with a limited number of obtained modes and a therefore non-squared mode shape matrix [6]. In a first step for applying this procedure, the modal coordinates $q_{r}$ are approximated, which can be represented the best (see Equation (5)).

(5) $$q_{r}={\left(\phi_{dr}^{T}\phi_{dr}\right)}^{-1}\phi_{dr}^{T}u$$

In this equation, $\phi_{dr}$ is the non-squared modal matrix and u are the displacements. After computing $q_{r}$ , Equation (5) can be used to calculate the energy contribution of each observed mode i , where $\omega_{i}$ is the angular frequency of the mode under consideration.

(6) $$E_{r,i}=\frac{1}{2}\omega_{i}^{2}q_{r}^{2}$$

Comparing the energetic contribution of each mode allows then to select the modes *M < S* with the highest contribution, which are then considered further for the shape sensing task [6,29].

Applications of the MM can be found in various studies in the literature. For example, it is applied by Valoriani et al. and Drachinsky et al. on the example of an aircraft wing [24,30] or by Esposito and Gherlone on the example of a wing box [6]. The application demonstrates that the MM can yield good shape reconstruction results with fewer sensors than iFEM. However, after an optimisation of sensor positions in the 2020 study of Esposito and Gherlone, it was still found that a total of 162 strain gauges was required [6]. Besides, a drawback of the MM is the need to perform additional simulations for the purpose of mode shape estimation, which increases the effort for the application. On the other hand, due to the use of simple matrix-based equations, the implementation is still straightforward, and for simple structures, it was proven to work with a realisable number of sensors [31].

#### 2.1.2. Physics-Informed Neural Networks for Shape Sensing

Artificial neural networks (ANNs) already showed great potential for shape sensing applications, as demonstrated in studies like Bruno et al. 1994 [17]. However, the data dependability and, in case of too little diversity in training data, also the generalisability of predictions were often found to be an issue in applications. To overcome this issue, physics-informed neural networks (PINNs), as introduced by Raissi et al. 2019, are a promising solution to overcome this issue [19]. In these models, prior knowledge of the problem is incorporated as soft constraints into the loss function for supervised transfer learning. These soft constraints include the governing physics laws, for example, expressed in partial differential equations, which the PINN learns to solve during training, making it easier to understand the connection between inputs and predicted outputs in terms of physical correctness. Furthermore, physics laws can be incorporated in the loss terms during training in the form of initial and boundary conditions, e.g., the Dirichlet boundary condition for mechanical problems. Resulting from this, labelled data are not necessarily required for PINNs as they are for conventional purely data-driven ANNs, leading to a less biased performance by the training data [19,32].

**Figure 1:**
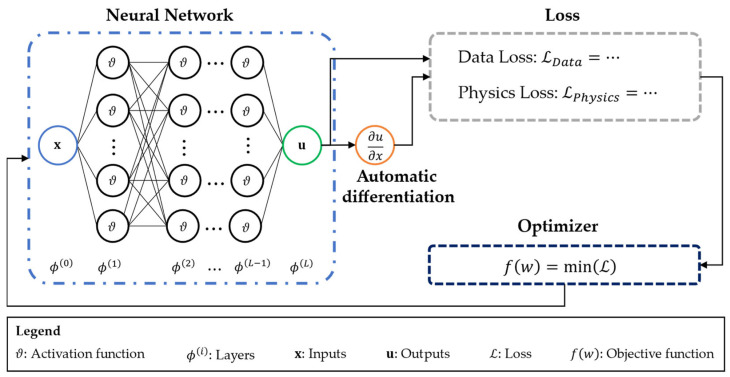
Sketch of the architecture of a PINN.

The basic principle for a PINN is shown in Figure 1, where the main components of a PINN, the neural network, the automatic differentiation, the loss, and the optimiser, are shown. In the beginning part, there is a neural network with *L + 1* layers, where the 0th layer is the input and the *L*th layer is the output layer. For each neuron of the hidden layers in between, an activation function ϑ is chosen and a weight *w_i_* is assigned. This enables the processing and transformation of data between layers, allowing the neural network to learn non-linearities between inputs and outputs. To include the physics laws, an automatic differentiation is then performed for the predicted outputs in dependence on the inputs. Therefore, the PINN learns to solve partial differential equations (PDEs). The predictions and results from the differentiation are then processed in the loss during training, resulting in a data loss component, as in purely data-driven ANNs, and the physics loss, which can consist of multiple components, depending on the considered problem. This loss serves as the basis for the optimiser, which aims to minimise it. For this purpose, the weights of the neurons in the hidden layers are adjusted in each training epoch. For more detailed information on PINNs and their implementation, see [33].

However, it is also possible to incorporate prior knowledge into a PINN, for example, in the form of training data from simulations or real-world experiments. Therefore, PINNs are distinguished into conventional PINNs as introduced by Raissi et al. [19]; data-assisted PINNs (DA-PINN), as described by Wang et al. [34], where training data are used to enhance the pure PDE-based training; and data-driven PINNs, as described by Yang et al. [35], where the training is purely based on training datasets and PDEs are incorporated only for the physical correctness.

Applications of PINNs for solving inverse problems, hereinafter referred to as iPINNs, as in the case of shape sensing, can be found in different applications in the literature. For example, Qui et al. introduced their iPINN called “SensNet” in 2023. This iPINN is developed to reconstruct the deformed shape of a two-dimensional (2D) or a three-dimensional (3D) part at the collocation points based on strain inputs from discrete sensor positions. For this purpose, three loss components are used in training: The data loss of predicted displacements to reference displacements, the boundary condition loss at clamped ends, and the strain loss of predicted strains based on the partial derivatives of the predicted displacements. The applicability is demonstrated on the example of a cantilever beam and was found to work very accurately [32]. Another application can be found in the paper of Yan et al., where iPINNs are used for shape sensing of a wing box of an aircraft. However, due to the more complex shape, they decided to use more than a single iPINN for better prediction accuracy [36]. The approach of different PINNs was also applied by Bai et al., where there were three different PINNs trained, one for each displacement component of a 3D problem. However, the study demonstrated that this approach to applying PINNs leads to accuracy issues when one PINN is trained more effectively than another, due to the presence of one displacement component over others in the data. Besides, these PINNs were not iPINNs, since they solved forward problems by predicting displacements based on known boundary conditions and loads [33]. In cases of unknown loads, with the primary objective of identification, the iPINN of Xu et al. stands out as a notable example in the literature. This work focuses on the problem of identifying external loads of structures, and the applicability is demonstrated on the example of a 2D tube cross-section with internal pressure as well as on a cantilever beam, where a high accuracy could be observed [37]. Besides this work, Go et al. presented an iPINN for shape sensing for 2D problems on the example of a plate. This iPINN has an architecture where the collocation points are inputs in neurons for each dimension (x, y) separately. Furthermore, sensor data are fed to the neural network with an input neuron for each sensor. As loss terms, five loss terms, e.g., one for the Dirichlet and one for the Neumann boundary condition loss, were used. The iPINNs performance was investigated for different numbers of sensors as inputs, where a dependence on the central position sensor in the middle of the plate was found. Furthermore, the influence of the presence of measurement noise was investigated, where the prediction accuracies decreased with higher noise amplitudes. Interestingly, the accuracy was found to be best with a few sensors in case of noise due to fewer sources for confusion of the iPINN. However, a drawback of this iPINN is the limitation to plate-like structures and only 2D deformations [20].

From the above-described studies, it becomes clear that iPINNs are suitable when it comes to high-accuracy shape sensing applications with less training data dependence than in conventional neural networks. Especially, the need for only a few required sensors makes them well-suited candidates for real-time shape sensing using WSNs [20,38].

### 2.2. Wireless Sensor Networks for Monitoring Structural Components

**Figure 2:**
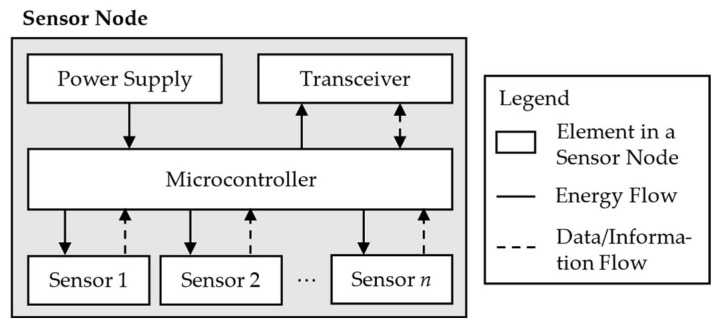
Sketch of a Sensor Node in a Wireless Sensor Network.

In the field of SHM, especially of large-scale components, WSNs are well-suited techniques [39,40]. These are sensor networks where one or more sensors are combined into a so-called sensor node, which consists of the sensors in case it is needed, analogue-digital converters and amplifiers, a microcontroller, a power supply, and an interface for data transmission and receiving [41]. A sketch of this principle is shown in Figure 2, where the flow of energy and data or information in the system is shown by the solid and, respectively, dotted lines. The microcontroller receives energy from a power supply, e.g., a battery. This energy is used to power the sensor or sensors for data acquisition. Consequently, the measurement data are transferred back to the microprocessor for processing. In case a conversion is needed, a converter and/or amplifier can be used in between. At the microprocessor, the raw data are processed and transferred in the form of information to the transceiver. Furthermore, the transceiver is powered by the microprocessor again [42]. However, depending on the application, the transceiver also receives data. This can be, for example, data packages from other sensor nodes for further transmission or data for synchronisation of sensor nodes from a server [43,44].

**Figure 3:**
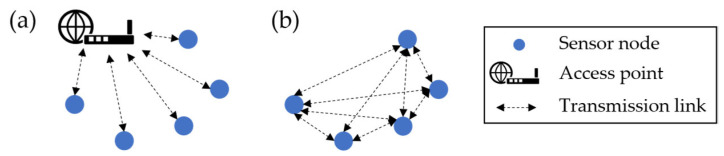
Two types of wireless sensor networks: (**a**) an infrastructured and (**b**) a non-infrastructured (based on [46]).

This already shows that there are different kinds of data transmissions in WSNs, which depend on the chosen type and architecture for the network. The literature, therefore, distinguishes two forms of WSNs: infrastructured and non-infrastructured WSNs. These two types are shown in Figure 3. In the case of an infrastructured WSN (see Figure 3a), the sensor nodes join an existing network, like a wireless local area network (WLAN). Resulting from this, all sensor nodes communicate with one access point, which forms a star topology of the network. Compared to this, non-infrastructured, and especially ad-hoc networks, are self-establishing, organising and adapting networks in which each participant can communicate directly with all other participants within range [45]. Therefore, data transmission protocols have a high importance [46]. Resulting from the chosen protocol, different forms of architectures can result in contrast to the infrastructured WSNs. Suppose nodes are defined as cluster heads, which sample data from an assigned set of sensor nodes before transmitting these data packages to a sink node [43]. However, in the following, only infrastructured networks will be considered further for the WSN application in this paper. Therefore, when referring to a WSN in the following, an infrastructured WSN is considered.

In the literature, various studies using WSNs in SHM can be found [47]. In the following, studies using a WSN for strain measurements, as it is necessary for shape sensing, will be presented. Especially for civil infrastructures, such examples can be found. For instance, Liu et al. 2021 implement a WSN incorporating multiple fibre Bragg gratings (FBGs) as fibre optic sensors to monitor strata deformation within a borehole [48]. Similarly, Taher et al. 2022 deploy a WSN equipped with custom-developed strain sensors for monitoring fatigue cracks in steel bridge components [49]. In another example, Herrasti et al. 2016 introduce a sensor node capable of wirelessly recording acceleration, temperature, and strain, demonstrated through its application on a wind turbine. The system is benchmarked against a wired alternative and achieves comparable results in vibration mode identification and environmental monitoring, offering a cost-effective solution [50]. Likewise, Tantele et al. 2016 present a WSN for evaluating the structural condition of a highway bridge, integrating three sensor nodes with strain, temperature, and wind sensors, and employing WiFi connectivity in field deployment. Data interpretation is supported through a graphical user interface (GUI). Beyond civil infrastructure, WSNs have also been explored in the aerospace sector [51]. For example, Wu et al., 2009a developed sensor nodes for strain monitoring on a carbon fibre reinforced plastic (CFRP) wing box, addressing reliable data transmission and routing strategies [52]. In a follow-up study, Wu et al. 2009b utilise these nodes in a structural test of a scaled aircraft wing. However, the sensor design is limited by the size of the transmission and protective units [53]. Another application of a WSN is described in [42], where strain gauges are connected with analogue/digital (A/D) converters to Arduino Nano 33 IoT microprocessors for the purpose of shape sensing using Ko’s displacement theory. However, the applicability was demonstrated, but without any optimisation of sensor and sensor node positions.

In all the presented studies, it becomes clear that there are either no shape sensing applications considered when a WSN is used for strain-based SHM or no sensor positions are optimised. Furthermore, a common approach for sensor and sensor node position optimisation is not considered, as pointed out in [13]. Therefore, this issue will be addressed in the following.

## 3. Methodology

**Figure 4:**
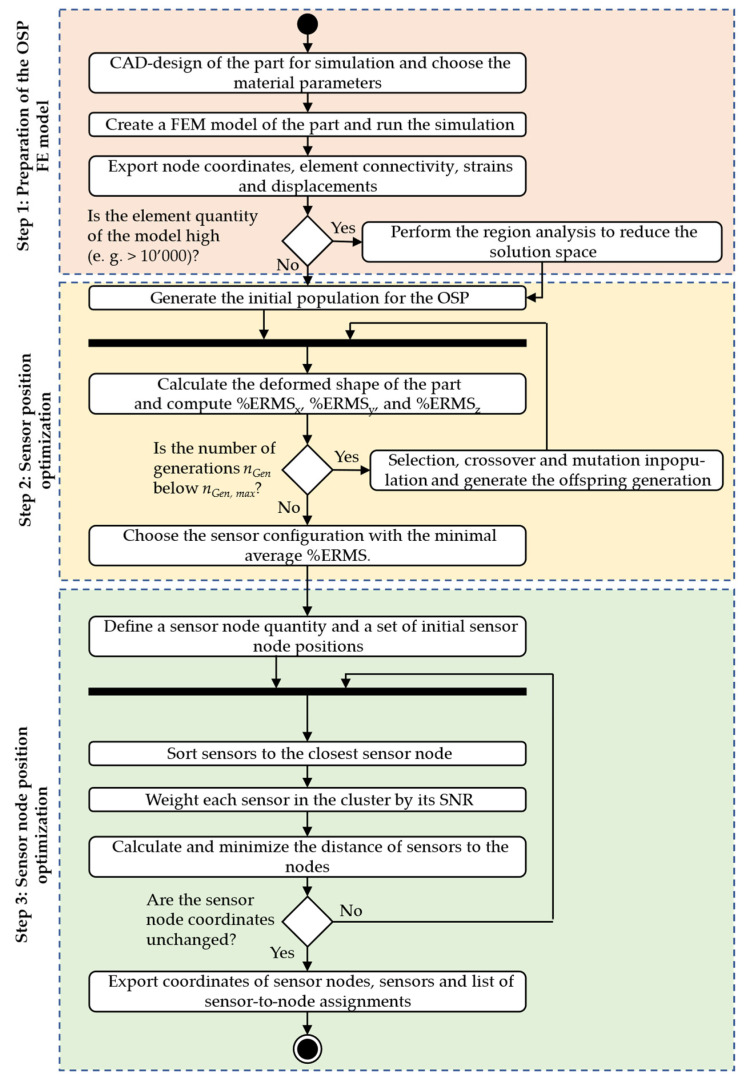
Methodology for the optimal sensor and sensor node placement with a three-step process.

The optimisation of sensor positions is part of various studies, for example, in the work of Esposito et al. [6]. However, further consideration of the derived sensor positions for a WSN is not emphasised enough in the literature. Only in [13] is a methodology presented for that purpose. In this study, however, only a methodology is proposed, which was mainly focused on an OSP for two different product lifecycle phases. But since the approach is promising and requires a practical verification, it is chosen for this study. Because only the application for shape sensing is considered here, the methodology is not used and adapted in its completeness. Therefore, it is described in the following with its implementation. An overview of the required steps is given in Figure 4 in the form of a process model in Unified Modelling Language (UML).

In the beginning, a FEM-based simulation is carried out. Therefore, the part under consideration has to be designed in a model using computer-aided design (CAD) first and is then imported into the FEM software. Material parameters have to be chosen as for the real part, which should be equipped with the WSN for shape sensing. Furthermore, a choice has to be made for the most suitable and representative load case, for which the sensor and sensor node positions should be optimised. After this step, the simulation can be submitted for computation. However, if a fine discretisation is chosen, a large solution space results for the OSP, since each element of the FE model can be considered as a placement option. This results in a large solution space, where optimisation algorithms struggle to find global optima, which results in suboptimal solutions [54]. To overcome this issue, the solution space can be narrowed by applying the so-called region analysis, proposed in [41]. Here, the region growing algorithm from image analysis is used to cluster elements from a FE model into regions by their strain values in dependence of the measurement system’s tolerance. This strategy was, for example, applied by Galfione et al. 2025 to the example of a BOOM structure of a satellite, where the FE model with more than 10’000 elements was no longer suitable for finding optimal solutions with small error tolerances for the objective function in the optimisation process [31].

After the FE model is generated, the data for the OSP is generated, and if necessary, the region analysis is performed. The OSP is started in the second step of the procedure. In the methodology applied in this paper, the optimisation is based on a genetic algorithm. Therefore, an initial population is generated in the beginning, where each individual describes a sensor configuration by the IDs of the elements or regions where a sensor is positioned, similar to the approach described in [55]. For each of the individuals, the deformed shape is then reconstructed based on the chosen shape sensing technique and the percentage root mean square error *%ERMS* is calculated for the three displacement components as shown in Equation (7).

(7) $${\%ERMS}_{i}=100\cdot \sqrt{\frac{1}{n}\cdot \sum_{i=1}^{n}{\left(\frac{u_{i}-u_{i}^{ref}}{u_{i,max}^{ref}}\right)}^{2}}$$

Here, $u_{i}$ is the displacement at the position *i* of all *n* positions in the FE model, and $u_{i}^{ref}$ is the corresponding reference displacement at this position. The difference between them is further weighted by the maximum displacement in the reference solution $u_{i,max}^{ref}$ for the considered displacement component.

After this is carried out for all the individuals in the population, it is checked if the current number of generations *n_Gen_*, does not exceed the maximum number of generations *n_Gen,max_*, which is defined before the optimisation process. If this is not the case, the best-suited individuals are identified by the lowest *%ERMS*, and crossover and mutation are performed to generate the next generation population, as in the case of the non-dominating sorting algorithm II (NSGA II) as a multi-objective algorithm [56]. This step is then carried out until the condition *n_Gen_ > n_Gen,max_* is fulfilled, so the objective functions $f_{i}$ in Equation (8) are fulfilled. From all considered individuals, the solution with the lowest *%ERMS* is then chosen as the sensor configuration for the shape sensing task.

(8) $$f_{i}=\min\left({\%ERMS}_{j}\right)\;with\;i=1,\;2,\;3\;and\;j=x,y,z$$

In the third step of the procedure, the position of the microprocessors as the centre of the sensor nodes has to be optimised, which is why it is described as optimal sensor node placement in the following. This step is carried out as described in [13] based on the ADD algorithm for a warehouse location problem [57]. In this case, the position of the microprocessor is the warehouse, which location needs to be optimized, and the “customers” to sort to the warehouse are the sensors in the already optimized configuration. Even though this is a heuristic approach, which might not lead to a global optimum for the solution, it is chosen because of the fast computation and the proven suitability for such problems in other applications in the literature. At the beginning of this step in the methodology, a maximum number of sensor nodes must be defined for the WSN to be developed. On this basis, an initial solution for the sensor node positions is generated. The sensors are then clustered to the nodes by finding the node with the minimal Euclidean distance to the corresponding sensor. Since the optimisation of node positions focuses not only on reducing the total distance to connected sensors but also on minimising cable length to limit signal degradation, too, the distance between each sensor and its associated node must be minimised. Accordingly, the objective function *F* defined in Equation (9) is used for the optimisation. Here, $\left(x_{i},\;y_{i},z_{i}\;\right)$ denote the coordinates of the currently considered sensor *i* and $\left(x_{i,Node},\;y_{i,Node},z_{i,Node}\;\right)$ their respective node positions.

(9) $$F=\underset{x,y,z}{\min}\left[\sum_{i}w_{i}\left(\left|x_{i}-x_{Node,i}\right|+\left|y_{i}-y_{Node,i}\right|+\left|z_{i}-z_{Node,i}\right|\right)\right]$$

Furthermore, a specific weight $w_{i}$ is assigned for each sensor, which is derived from the individual signal-to-noise ratio (SNR). Sensors with lower SNR are assigned higher weights to prioritise minimising their connection distances. This strategy helps mitigate further signal degradation by positioning nodes closer to more vulnerable sensors.

The optimisation process iteratively updates node locations inside an allowed solution space, e.g., considering only specific faces of parts, based on this criterion until the positions converge. At this point, they are considered optimal for the current sensor layout. If this is the case, the whole optimal sensor and sensor node placement is finished, and the results need to be exported. Therefore, the sensor and sensor node positions are saved along with the assignments of sensors to the nodes so that this can be used for installations afterwards.

## 4. Case Study on the Application

In the following section, the above-described methodology is applied in a case study, using a Python-based implementation of it. Therefore, the part and load case considered in this study will be described, as well as the resulting FE model for data generation. Afterwards, the optimal sensor and sensor node placement is described with the results. This is then used for a real-world application on the demonstrator on a test bench.

### 4.1. Demonstration Part, Load Case and Simulation

**Figure 5:**
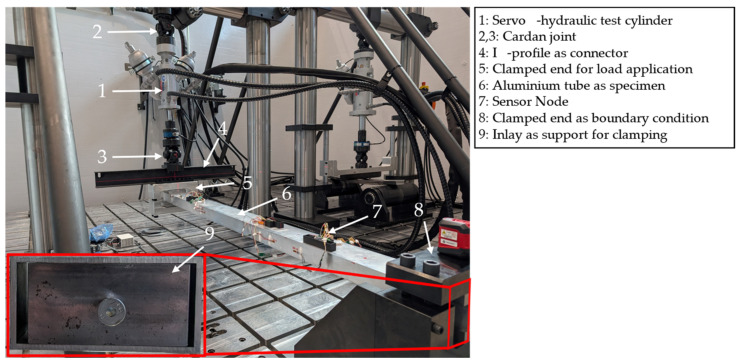
Setup of the tubular demonstration part under bending load applied by a servo-hydraulic test cylinder and with a clamp on the other end on the multi-axial, dynamic test bench.

To demonstrate the applicability of the methodology, a first demonstration is carried out on an aluminium part (EN AW 6060-T66 with E = 69 GPa and ν = 0.33) under bending load. In Figure 5, the demonstrator in the form of a rectangular tube is shown in the test setup at the test bench in the load case of this study. The tube has an outer cross-section of 120 mm × 60 mm and a thickness of 4 mm. In total, the tube has a length of 2000 mm. However, it is fully clamped on one end for a length of 120 mm. To avoid deformations due to compression while clamping, an inlay made of steel is used, which supports the tube for the whole 120 mm length.

On the other side, the load is applied by a servo-hydraulic test cylinder with a maximum static force of 10 kN, a maximum way of 20 mm, and a maximum frequency of 300 Hz. To apply the load on the demonstrator, another component for clamping is connected to the test cylinder. Here, the tube is clamped for an additional 120 mm to make sure the displacement is correctly applied. Therefore, a free length of 1880 mm is left for the deformation of the part. As shown in Figure 5, the test cylinder is not directly connected to the part but by a cardan joint to an I-profile. On the one hand, this design prevents lateral forces from acting on the test cylinder, and on the other hand, it enables loads to be applied on the demonstrator not only in the form of pure bending but also in the form of bending with superimposed torsion by repositioning the test cylinder. However, in the following, only the case of pure bending is considered, as shown in the setup.

**Figure 6:**
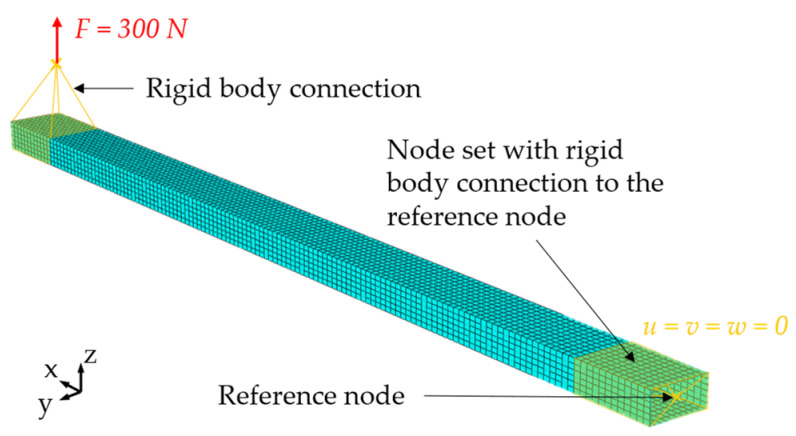
FE mesh of the aluminium tube with marked areas for node sets with rigid body connections to the reference points for load applications and boundary conditions definition.

Resulting from this real-world case, an FE model is derived and set up in Abaqus. A sketch of this model is shown in Figure 6. The tube is modelled as a shell structure due to the thin-walled design. In this case, S4 elements without reduced integration and an edge length of 10 mm are used, resulting in a total of 4480 elements. For the definition of the boundary conditions and the loads, two reference nodes are defined and connected to the node sets at the component by rigid body elements. On the reference node, the translational degrees of freedom (DoF) are blocked $\left(u\;=\;v\;=\;w\;=\;0\right)$ and the node is connected to all nodes in the area x ≤ 120 mm. Only the translational DoF are blocked, since it is hard to control the rotational DoF in real test setups. On the other end, a reference node is defined above the part, where the cardan joint is positioned. On this node, all DoF are free, and a concentrated force *F* = 300 N is defined in the *z*-direction. This reference node is connected to a set of nodes in the range of 1880 mm ≤ x ≤ 2000 mm so that the load is equally applied in this area. Since the MM should be applied in the following, the analysis is carried out for the natural frequencies as well, to obtain data for the modal coordinates and strains. Here, a max. frequency of 10 Hz is assumed since the tests will be carried out with only low frequencies to avoid damage to the WSN in the prototype application. The data from this simulation is then exported in a *.txt file for later processing in the OSP.

Besides the MM, an iPINN should be used for the shape sensing task. Due to the simpler implementation by the consideration of training data and then by only ensuring correctly implemented partial differential equations, a data-driven iPINN (DD-iPINN) is considered in the following. In the following, this DD-iPINN will only be referred to as iPINN due to the shorter abbreviation. Therefore, further training datasets are required. These are generated using a Python script for a Monte Carlo simulation with 100 iterations of the aforementioned FE simulation with a random variation of the applied force *F*. For this purpose, a normal distribution with *µ* = 300 N and *σ* = 50 N is defined, which ensures that the data are similar to the considered load case for the OSP but still with enough variance to avoid overfitting of the iPINN model. This data are also automatically exported in the form of *.txt files.

### 4.2. Optimisation Results for Sensor and Sensor Node Positions

**Figure 7:**
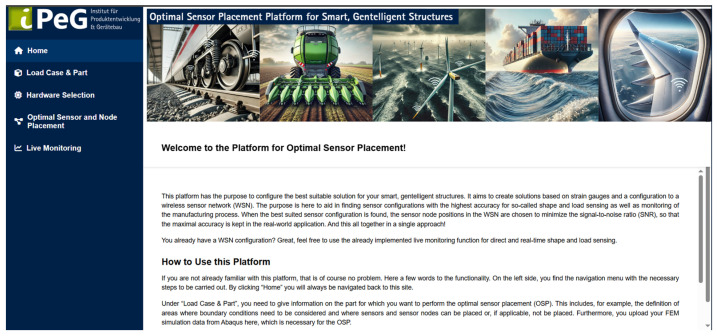
HTML interface for the tool for optimal sensor and node placement for shape sensing.

To carry out the optimisation of sensor and sensor node positions, a Python implementation of the methodology described in Section 3 is used. The implementation is realised with Python Flask and an HTML interface for user interaction. This interface is exemplified in Figure 7, using the “Home” tab as an example, which provides an introduction to this tool.

**Figure 8:**
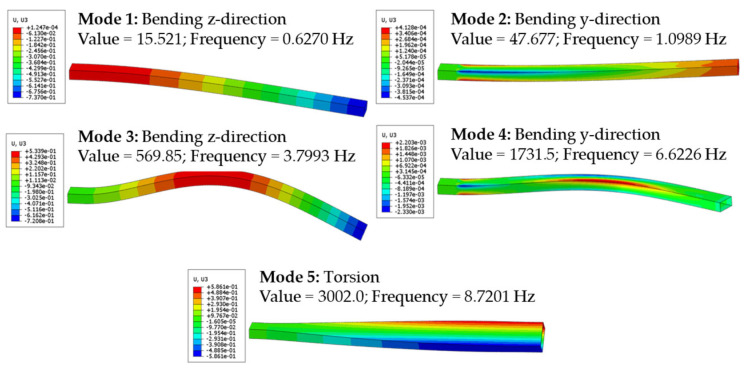
Mode shapes of the aluminium tube for 10 Hz frequency and exemplary colour plots for the z-displacements *w*.

It should be noted here that the OSP is only carried out for the MM, and the resulting sensor configuration is then used for the application of the iPINN as well. This is conducted because of the architecture of the chosen iPINN, which is similar to the architecture presented by Go et al. 2025 [20] and which can only be trained on predefined sensor positions. However, the architecture and training are presented in detail after the description of the OSP and the results. In this implementation, the first step is to load the simulation data for processing in the “Load Case & Part” tab. Here, the Abaqus *.inp file and the exported *.txt files with the simulation results are processed for further use. It starts with analysing the number of elements in the model to see if it is necessary or recommended to reduce the solution space by applying the RGA4FEM. However, in this study, this is not necessary due to the number of elements with less than 10’000. Therefore, the exported *.txt files are further processed to generate the matrices $\phi_{s}$ and $\phi_{d}$ for applying the MM. In the case of the above-described load case and the simulation, five modes are observed, which are shown in Figure 8. These result from a simulation with frequencies of up to 10 Hz, which are oriented on the experiments to be carried out, where higher frequencies are not used to avoid damages to the WSN.

After these steps, the “Hardware Selection” tab of the Python implementation is used to make some further choices influencing the optimal sensor and node placement. Therefore, it is necessary to specify which types of sensors should be used. In the case of this tool, it can be chosen between single linear strain gauges, half bridges, and strain rosettes, since the implementation is strain gauge specific. For this study, the single linear strain gauges are chosen to reduce the amount of required hardware. This choice allows for the measurement of only one strain component, rather than the three possible components in the plane. However, since the considered pure bending load case results in mainly strains along the parts’ length, the shape sensing accuracy should not be negatively influenced. Besides the sensor choice, the type of microprocessor must be selected, as it determines the number of sensors that can be clustered to a sensor node based on the available pins. In case of the implementation, it can be chosen between an Arduino Nano 33 IoT with 14 digital pins, a Raspberry Pi Pico W with 26 digital pins, and an ESP32-C03 with 22 digital pins. Since a primary study already successfully used a sensor node with an Arduino Nano 33 IoT [42], this microprocessor is chosen here as well due to the hardware availability. As the last parameter on this tab, the user must specify the expected level of measurement noise for the configured measurement system. Therefore, the noise amplitude has to be typed in and is set to $a_{Noise}$ = 2 µm/m in this study based on the primary study’s measurements.

Following these hardware selections, the OSP can finally be started in the “Optimal Sensor and Node Placement” tab of the HTML user interface. However, before this, the last settings for the optimisation algorithm have to be made. This includes, first of all, the definition of areas to be excluded from the OSP. For example, the clamped areas for the boundary condition and load application are excluded here so that sensors can be placed only in the area with 120 mm < x < 1880 mm of the aluminium rectangular tube. Therefore, from all 4480 elements as placement options, only 3904 remain available for placement. Furthermore, it is possible to define whether only the top or only the bottom surface areas should be taken into account. However, this is only set for the sensor node placement to make the hardware more straightforward to install. Besides, it is necessary to set the parameters for the genetic algorithm, since the NSGA-II algorithm is applied. Therefore, the max. number of generations is set to 50, as well as the number of individuals per generation. These numbers are chosen from primary studies, where increasing the number of individuals per generation and the number of generations did not result in better solutions but led to high computational costs. As the last parameter, the number of sensors is defined to be ten linear single strain gauges, and therefore, the number of microprocessors is set to three, allowing for the assignment of all sensors to nodes. This choice for the sensor quantity is made because of the available sensors for these tests and the size of the part. More sensors might lead to more accurate results but would result in more installation costs. Due to the required additional hardware, it is not sensible to use more strain gauges, since the installation space is also limited on this part. Since the sensor quantity *S* is already higher than the number of mode shapes *M* (see Figure 8), the criterion of Bogert et al. [29] is not applied. Therefore, all five mode shapes depicted are considered, even though an analysis showed that the energy contribution of modes 1 and 3 is over 99 %, which makes them especially relevant for the shape sensing application.

Building on this, the OSP is set up with these parameters for the optimisation algorithm and the matrices to apply the model method. It is then started in a Python implementation using the pymoo library with its NSGA-II implementation [58]. Therefore, the optimisation is carried out in a loop, where each sensor configuration as an individual of each generation is used for shape sensing with the MM. Based on the *%ERMS* for the three displacement components, the fitness is then evaluated, as described by the objective function in Section 3. However, this evaluation is carried out with two approaches. The first approach, carried out for the first half of generations, is the crowding distance-based evaluation. This allows a more expansive search space with a lower risk of being stuck in a local instead of a global optimum. After exploring the solution space in this wide way, the selection is switched to a binary tournament-based approach for the second half of generations, where only the two best individuals are selected for further reproduction. By doing so, the decision for the best individuals becomes a lot stricter with the potential to find individuals very close to an optimum while having faster convergence, which, however, would increase the risk of finding a local instead of a global optimum if applied too early. Both approaches are implemented in the pymoo library and are used as they are. For details on both approaches, see [56,59].

The choice for the overall best individual for the sensor configuration to be realised is then based on the calculation shown in Equation (10). Here, the Euclidean distance from each individual to the best possible solution with *%ERMS_x_ = %ERMS_y_ = %ERMS_z_ =* 0 is calculated, and the solution with the minimal value is chosen.

(10) $$\%ERMS=\sqrt{{{\%ERMS}_{x}}^{2}+{{\%ERMS}_{y}}^{2}+{{\%ERMS}_{z}}^{2}}$$

**Figure 9:**
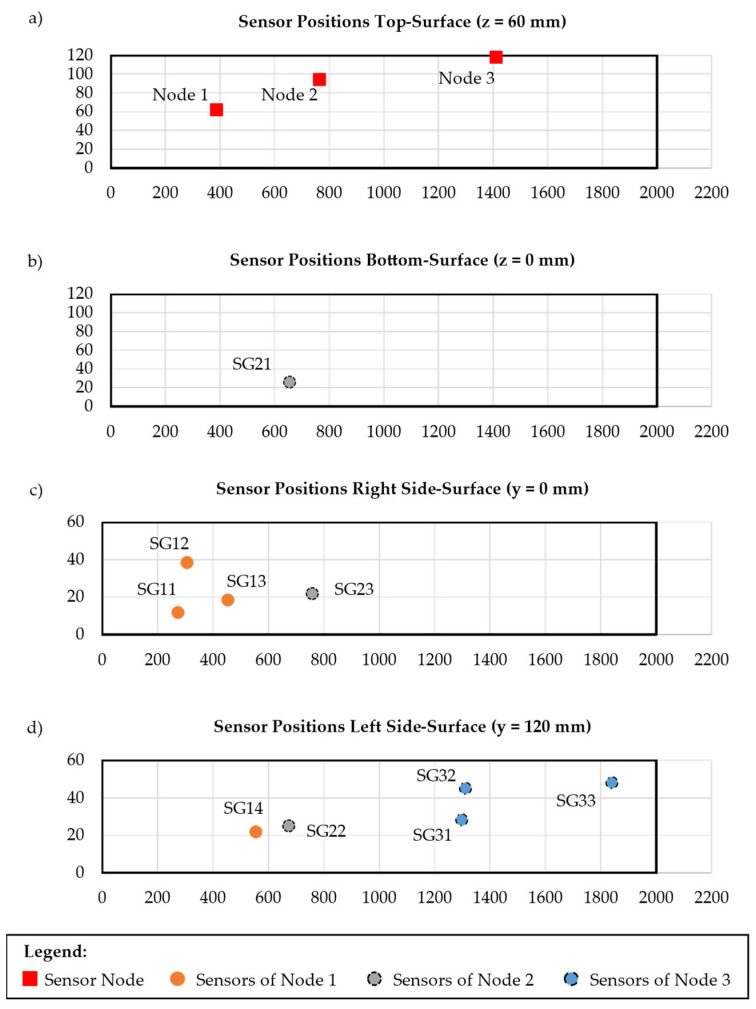
Sensor configuration and position of the sensor nodes resulting from the optimisation for the four faces of the rectangular tube with sensor IDs used during the test with (**a**) the top, (**b**) the bottom, (**c**) the right side and (**d**) the left side face.

From this procedure, the sensor configuration depicted in Figure 9 is selected for realisation, which results in an accuracy of *%ERMS_x_* = 3.35 %, *%ERMS_y_* = 4.59 %, and *%ERMS_z_* = 1.78 %.

Due to the comparatively small displacements and since the accuracy of shape reconstructions with the MM is challenging to obtain below 5 %, the results are very promising. The relatively high error for the *x*- and *y*-directions results from the smaller displacements in these directions, which occur because of the pure bending load case. However, since the NSGA-II aims to minimise the error for all displacement components, this solution is preferred to ones with a smaller *%ERMS_z_* but a lot higher *%ERMS_x_* and *%ERMS_y_*.

Based on the chosen sensor configuration, the optimal sensor node placement is carried out as well. For this purpose, the strains at the sensor position from the FEM simulation, as well as the user-defined noise amplitude $a_{Noise}$ , are used to calculate each sensor’s SNR for the weighted distance calculation in this third step of the methodology (see Section 3). For this calculation, Equation (11) is used, where $a_{Measurement}$ denotes the amplitude of measured strains.

(11) $$SNR\left[dB\right]=20\cdot \log_{10}\left(\frac{a_{Measurement}}{a_{Noise}}\right)$$

The resulting SNR values for each sensor can be found in the Appendix A of this paper. Since it was chosen to position sensor nodes only on the top surface of the part, only these 1220 elements are considered as placement options. If this constraint had not been applied, the risk for placements on the sides would have been very high due to the already strongly clustered sensors on these faces of the part. This would, e.g., happen for Nodes 2 and 3 with a high probability. For Node 1 there might be a risk of a position on the bottom surface as well, since the assigned sensors are positioned on different sides. However, the assignment of sensors to nodes would also change, so it is difficult to interpret the placement. Therefore, the sensor node layout results as shown in Figure 9a. The assignment of the strain gauges to these sensor nodes is furthermore marked in Figure 9 by the different colours of the sensor positions. This layout is used for the installation of the test, as described in the following Section 4.3.

Using the resulting sensor configuration, the iPINN is trained for the real-world application with the generated FEM training data sets. Therefore, the iPINN architecture is used as published in [60] and described in [38] as a fully connected neural network (FNN) with five hidden layers with 64 neurons each. As inputs, this iPINN receives colocation points *x*, *y*, and *z* as input vectors in three different neurons. Additionally, the sensor data from the single strain gauges *S_i_ = {x_i_, y_i_, z_i_, ε_xx_}* with only strain measurements in the *x*-direction is fed as inputs with one neuron for each sensor. This results in a total of 13 input neurons in this case. For the outputs, three neurons are used, where each neuron yields one displacement component vector for all the collocation points. All layers use the rectified linear unit (ReLU) as the activation function, since it allows better gradient propagation, which is essential for learning to solve partial differential equations as iPINN, and a linear activation function for the output layer. This architecture is derived from primary tests with different configurations, but it worked best for the present case. For incorporating training data as well as physics laws into the training, the loss terms in Equations (12) to (14) are utilized.

(12) $$L_{Data}=\frac{1}{3}\sum_{i=1}^{3}\left[\frac{1}{n}\cdot \sum_{j=1}^{n}{\left(u_{ij}^{pred}-u_{ij}^{ref}\right)}^{2}\right]$$

(13) $$L_{BC}=\frac{1}{3}\sum_{i=1}^{3}\left[\frac{1}{n_{BC}}\cdot \sum_{i=1}^{n_{BC}}{\left(u_{ij}^{pred}\right)}^{2}\right]$$

(14) $$L_{\varepsilon }=\frac{1}{3}\sum_{i=1}^{3}\left[\frac{1}{n}\cdot \sum_{j=1}^{n}{\left(\varepsilon_{ij}^{pred}-\varepsilon_{ij}^{ref}\right)}^{2}\right]$$

The data loss $L_{Data}$ is calculated as the mean of the mean squared error (MSE) between the predicted displacements $u_{ij}^{pred}$ and the reference displacements $u_{ij}^{ref}$ for all collocation points of all three displacement components. The boundary conditions of the clamped end of the tube are considered by the loss term $L_{BC}$ , where the average of the MSE of all three displacement components to the boundary condition of u=v=w=0 is calculated. The last loss component is the strain loss $L_{\varepsilon }$ , which ensures the physical correctness of predicted displacements in terms of the PDE connection of strains, displacements, and the collocation points as inputs. This composition of loss terms is inspired by the proposed PINN architecture of Qui et al. [32], and all three losses are combined in a total loss as shown in Equation (15).

(15) $$L=L_{\varepsilon }+L_{u}+L_{BC}$$

The iPINN is trained on the generated training data from the FEM simulations, using a two-step training process. First, the adaptive moment estimation (Adam) optimiser is used because it is well-suited for problems that are large in terms of data and/or parameters [61]. It is applied for training until the total loss, which is combined from the data loss, the boundary condition loss, and the physics loss in the form of the strains as partial derivatives of the predicted displacements, is below a threshold of $L_{total}\;=\;5\cdot 10^{-4}$ . This value proved to be in a good balance between a too-short training and an unnecessarily long training with high computational effort and a higher risk of overfitting. After this step, the Limited Memory Broyden–Fletcher–Goldfarb–Shanno (L-BFGS) optimiser is used for fine-tuning of the weights inside the iPINN. This whole training procedure is based on the description of the iPINN presented in [20]. After the training is finished, the iPINN is saved as *.keras model and is then available for the application in the live monitoring on the test bench as the last step of this case study.

### 4.3. Application on the Real-World Demonstrator on the Test Bench

**Figure 10:**
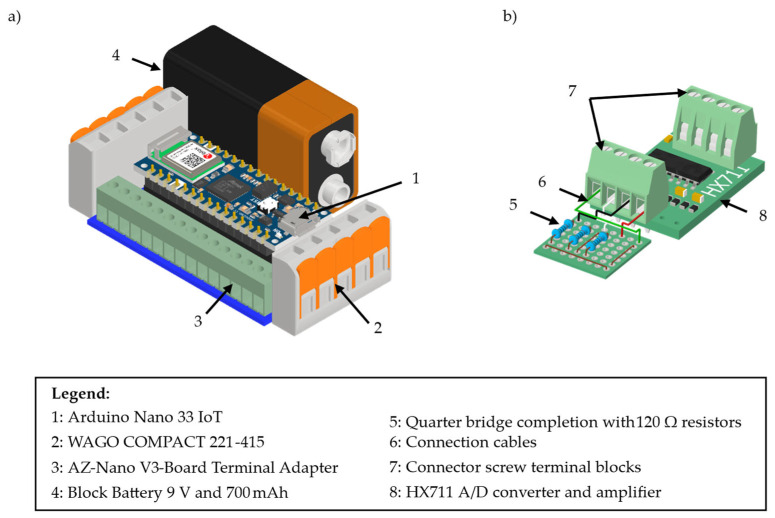
CAD model of the sensor node components with (**a**) the microprocessor, connectors, and a power supply and (**b**) the quarter bridge completion for single strain gauges and an A/D converter and amplifier.

The sensor and sensor node configuration resulting from the previously described optimisation is realised on the demonstration part described in Section 4.1. Therefore, the sensor nodes are manufactured with the following components. For the microprocessors, Arduino 33 IoT is chosen because it is small, has a large open-source platform that is easy to program, and the WiFi Nina module is well-suited for infrastructure-based WSN applications. The microprocessors are stacked on a terminal board of type AZ-Nano V3 as an adapter with screw terminal blocks for cable connections. To allow more than one sensor to be easily connected to the Arduino, the ground contact and the 3.3 V voltage contact are each connected via a cable to a WAGO COMPACT 221-415 five-pole connection clamp. Due to the five poles, it is possible to connect up to four sensors for voltage provision. As a power supply, a 9 V block battery with 700 mAh is chosen, which allows up to 12 h of continuous measurements and data transmission for four sensors working in parallel. To connect this battery to the microprocessor, a cable with a contact suited for the battery on one end and a Micro-USB contact on the other end is soldered. The resulting sensor node is shown as a CAD model in Figure 10a. The realised version of this sensor node is then assembled with a 3D-printed housing to keep the components in the desired positions and to isolate them from the electrically conductive aluminium tube.

These sensor nodes are connected with further cables and the screw terminal of the adapter board to the A/D converter and amplifier of type HX711, which is depicted in Figure 10b. However, since the choice was made for single-strain gauges in the OSP, it is not possible to connect them directly to the input contacts of the HX711, since it is designed to receive signals from a Wheatstone bridge. Therefore, a quarter bridge completion is designed and realised with 120 Ω resistors, suitable for the 120 Ω strain gauges, which are soldered on a perforated board.

After these designs are finalised and realised, the installation is carried out on the rectangular tube. As sensors, HBM 1-LY11-6/120 strain gauges are glued to the aluminium rectangular tube at the desired positions, as shown in Figure 9. The quarter bridge completions and HX711 are then installed as close as possible to each sensor to avoid negative influences on the analogue signal coming from the strain gauge. By doing so, it is ensured that the signal is converted to a digital, amplified one, which is more robust against environmental influences, e.g., due to temperature changes. The sensor nodes are then placed on the top surface of the part at the desired positions, and the cable from the HX711 is connected to the terminal adapter. Since the screw terminal blocks fasten the cables, measurement noise resulting from moving cables is reduced in comparison to plug connections, which was investigated in previous tests with JST-XH connector plugs. The housing containing the sensor nodes and quarter bridge, along with the HX711, is then glued to the surface of the tube. Furthermore, the cables connecting sensors and all the other components are glued to the tube as well to avoid noise from vibrations during the tests.

**Figure 11:**
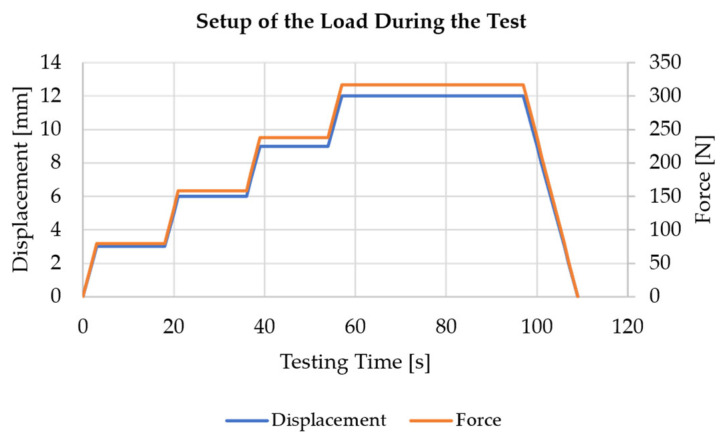
Test program with gradually increasing load and plateaus for the measurements.

Following this preparation, the aluminium tube is mounted on the test bench and aligned using a cross-line laser with a spirit level and additional angle measurement. The load is then applied following the specified test program shown in Figure 11.

In this setup, the load is applied gradually to allow measurements to be taken with sufficient time to compare specific, predefined maximum displacements. Therefore, plateaus exist for different loads. Even though Figure 11 shows both the displacement and the force applied by the test cylinder over time, the test is performed with displacement control only. This is chosen because the loads are low in comparison to the max. force of the servo-hydraulic test cylinder, so that the force signal is hard to distinguish from the noise, which complicates force control. This test program is run three times, and the data are logged by the sensor nodes and visualised in a strain-over-time plot on the “Live Monitoring” tab of the interface.

**Figure 12:**
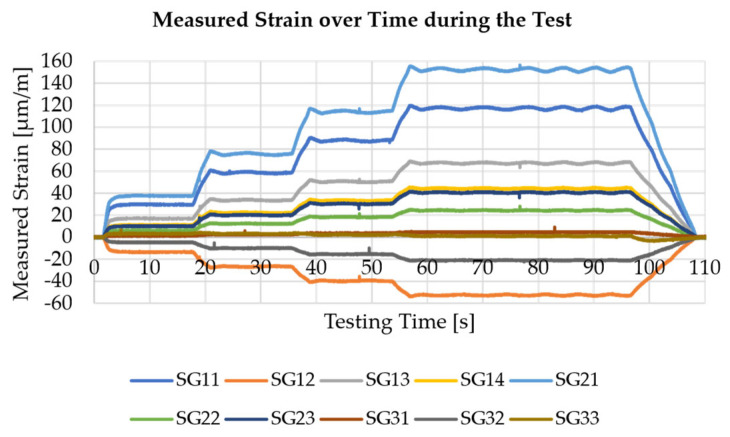
Measured strains of the ten strain gauges (SG) over the time of the test running.

A plot of the measured strains for one of the three test runs is shown in Figure 12. For this purpose, it is necessary that the PC, on which the server is running, is connected to a specific WLAN, since it is important to use the correct IP address with the server for sensor nodes. Besides, after all data packages from all sensor nodes arrive at the Flask server, the shape sensing task is carried out for all timesteps included in the measurements. However, before doing so, it is necessary to carry out a calibration, since the signal of some sensors shows inverted signals, which might result from incorrectly soldered cables. Following this calibration and starting the measurement, real-time shape sensing with a visualisation of the deformed shape in a PyVista plot beside the plot on the HTML interface is possible.

For a visual comparison of the reconstructed displacement field to the expected field from an FEM simulation, the case of the 12 mm displacement, or, respectively, the 316.88 N force as load, is investigated in the following. In Table 1, the strains at the sensor positions are shown for this comparison after the calibration of sensor node measurements. From the comparison, it already becomes clear that there is a discrepancy between the measured strains and the ones obtained in the simulation for all sensors.

For all sensors 11 to 31, the discrepancy is about 30 % to 35 %, which shows a clear deviation for all of them. This more or less systematic difference indicates that a discrepancy between the FEM model and the real-world test is present. A possible influence in that matter is the difficulty in controlling the boundary conditions, especially the rotational degrees of freedom. Furthermore, due to the cardan joint mounted to the test cylinder, the load is not applied completely vertically to the part, as in the FEM model. This is a second influence leading to discrepancy. However, for SG 32 and 33, the deviations are even higher. A possible reason for this problem is the low SNR due to the low strain values closer to the load application end of the part (see Appendix A). Besides, possible influences are resulting from manually gluing the strain gauges to the part, which results in unequal bonding to the part. Nevertheless, these values are considered in the form in the following for the shape sensing task.

**Figure 13:**
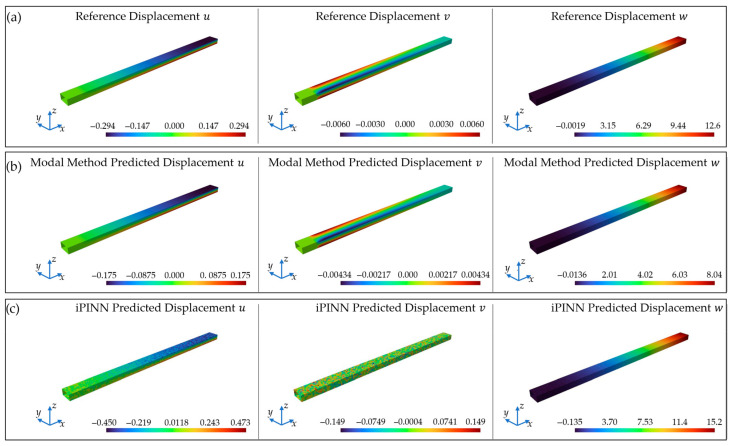
Comparison of the displacement fields for the displacement components *u*, *v*, and *w* for (**a**) the FEM simulation with 12 mm displacement load, (**b**) the MM field reconstruction, and (**c**) the iPINN field reconstruction from the WSN measurements.

In Figure 13, the plot of the three displacement components *u*, *v*, and *w* is shown in comparison to the FEM reference solutions when the MM and the trained iPINN are used. In this comparison, the high accuracy for the displacement field reconstruction with the MM is observable in Figure 13b.

In contrast to the FEM reference solution, the displacements are slightly underestimated, with only minor deviations of *%ERMSx* = 23.66%, *%ERMSy* = 12.21%, and *%ERMSz* = 16.34%. These values are higher than in the OSP results, which is because of the lower measured strains than the ones from the simulation as well as the presence of measurement noise and a control-initiated oscillation of the applied load, as shown in Figure 12. However, the shape reconstruction still works robust in terms of displacement distributions. This fits simulation-based investigations on the robustness of the MM, e.g., in the context of material uncertainties as in [26].

On the other hand, it becomes clear that the iPINN leads to worse results with comparatively low accuracy and errors of *%ERMSx* = 11.01%, *%ERMSy* = 58.8%, and *%ERMSz* = 37.3%. Especially the displacement component *v* in the *y*-direction exhibits a non-physical displacement distribution, indicating that it struggled to learn the structural behaviour for this component. Furthermore, it becomes clear that the displacements are significantly overestimated. A possible reason for the worse predictions than those from the MM lies in the measurement data. Even though PINNs are found to be less dependent on training data than conventional, purely data-driven ANNs, there is still some dependence on training data [38,62]. Since the training data comes from noise-free FEM simulations, the iPINN might struggle with the signals in the presence of noise, which therefore show stronger deviations from the initially seen data. Furthermore, it is worth noting that the displacements, particularly in the *x-* and *y*-directions, are very small and more challenging for the iPINN to learn due to the few differences. This issue, on the other hand, is not present for the input data in the form of the measured strains, since these are scaled to be in an interval of [0, 1] by applying Equation (16). Here, *ε_xx,min_* and *ε_xx,max_* are the minimal and maximal present strains *ε_xx_* from all training datasets, and *ε* is the strain value to scale. However, even though the iPINN is outperformed by the MM in terms of accuracy, in this study, both techniques proved to be applicable with very fast, real-time computations after data packages from the sensor nodes arrived. Therefore, both the methodology and the realised WSN are verified in terms of functional fulfilment.

(16) $$\varepsilon_{xx,scaled}=\frac{\varepsilon -\varepsilon_{xx,min}}{\varepsilon_{xx,max}-\varepsilon_{xx,min}}$$

## 5. Discussion

The presented methodology and the WSN used in the practical case study proved the usability of an approach for not only optimising sensor positions but also sensor node positions for maximal accuracy in shape sensing. Although the use of WSNs in shape sensing applications is not emphasised in the literature, it has been proven feasible with distributed sensor nodes on a specimen using a prototype without self-designed PCBs. However, there are still some aspects to be discussed regarding the methodology and the results in a critical assessment.

The results of the OSP and the shape sensing, especially for the MM application, showed high accuracy results for the load case for which the sensor and sensor node configuration is optimised. However, for real-world applications besides the test bench setup, more than one load case may occur, for example, in the case of an aeroplane facing turbulence. In such a case, where not only bending is considered, the accuracy might be significantly reduced, resulting in possibly incorrect decisions based on the reconstructed fields. Therefore, an enhancement of the methodology to the consideration of different kinds of load cases into the OSP as presented by Galfione et al. 2025 [31] would be sensible. Regarding the iPINN, this issue can be more easily addressed by incorporating training data from various load cases to facilitate predictions for other load cases beyond bending. However, in the application of an iPINN for this real-world shape sensing application, there is still potential for improvements due to the comparatively low accuracy. For example, using the acquired data from tests with measurement noise as training data can incorporate more realistic data into the training. On the other hand, it is worth noting that the training data may not only address the observed prediction accuracy issue. Another consideration is that the sensor positions used in this study might be optimal for the iPINN, even though they were selected for the MM. The current architecture of the iPINN is not applicable for an OSP without training, as it incurs high computational and timely costs for each sensor configuration proposed by the optimisation algorithm. This highlights a need for improvement in further investigations.

Another aspect to discuss regarding the results is the need for further investigations of the methodology on larger components. Although the demonstration part is well-suited for initial functional verifications of the three-node-based WSN, a larger demonstration part could further investigate the influence of non-optimal sensor node positions. In case of longer distances between sensors and the microprocessors, the impact of signal degradation and increasing SNR could be investigated further, which was not possible in the presented study.

Besides, some aspects of the methodology require critical evaluation as well. Although the methodology proved to be effective and presented a promising approach to combining optimal sensor and sensor node placement, it has some potential issues regarding the optimality of the solutions. First of all, it has to be noted that the ADD algorithm is a heuristic algorithm that always has the potential of yielding local instead of global optima. Since the sensor positions mainly influence the shape sensing accuracy, and the influence of cable length should be comparatively low in the present study, this influence is negligible. However, since the methodology aims to yield an optimal solution for both the sensor configuration and the sensor node positions, the approach with two separate steps might not result in an overall optimal solution. However, because this study is a first step towards a combined approach for both topics, this issue can be addressed in subsequent works that build upon it.

Lastly, a non-negligible issue and influence on the results might come from the hardware used in this study. The noise level is comparatively high, which shows a bad influence on shape reconstruction accuracy. One source for these influences on the signal quality might be the number of interfaces between the hardware components, e.g., with the cables connecting the quarter bridge completions and the HX711 A/D converters and amplifiers. Besides, the self-soldered quarter bridge completions also influence signal accuracy due to possibly varying resistances between the resistors of the Wheatstone bridge. Furthermore, the manual performance of gluing strain gauges to the parts results in unequal bonding of the strain gauges, which is also a possible influence on the measurement accuracy. Besides, it was found that the temperature of the single strain gauges increased during the test until it reached a constant value after ca. 30 min. This rising temperature comes from the current running through the strain gauges, and due to the missing temperature compensation of the other resistances, another influence on measurement accuracy is resulting. In the case of the robust MM, it appears to be less critical than in the case of the iPINN. However, if an iPINN is used, it is crucial to minimise the hardware influence to achieve maximal accuracy.

## 6. Conclusions and Future Works

Shape sensing and inverse methods in general, as part of SHM, have shown an increasing interest in recent years. For data acquisition using such methods, WSNs are particularly attractive for remote monitoring, especially of large-scale structural components, as they eliminate the need for lengthy cables, thereby reducing the risk of signal quality loss. However, WSNs for shape sensing, consisting of more than one sensor node, are not emphasised enough in the literature. Furthermore, there is currently a lack of literature addressing physics-informed neural networks (PINNs) for practical applications. Lastly, studies presented in the literature often only carry out an optimal sensor placement but do not consider optimisation of sensor node positions for a WSN as well. These three issues are therefore addressed in the present paper. Thus, a methodology is presented, which consists of three steps: Preparation of the FEM model for optimal sensor placement, sensor placement optimisation, and sensor node placement optimisation. This methodology allows to carry out an optimisation of sensor positions for the highest accuracy of the displacement field reconstruction as well as the optimisation of sensor node positions regarding the clustering of sensors to the nodes and the positions to ensure maximal data accuracy regarding the overall signal-to-noise ratio (SNR). To make the methodology applicable, it is implemented into a Python-based tool, where a Flask development server hosts an HTML interface to allow user interaction for setting up the optimal sensor and node placement.

To demonstrate the applicability of the methodology, a case study is conducted using a real-world test setup on a test bench with a 2000 mm long rectangular aluminium tube under bending load. For this tube, an FEM simulation is carried out in Abaqus Standard/Explicit 2024 to generate data for the optimisations and to train the PINN for solving inverse problems (iPINN) to use in this study. With the Python implementation of the NSGA-II algorithm of the pymoo library, the positions of ten single-strain gauges to be used are optimised. The resulting sensor configuration is then used to optimise the positions of the sensor nodes with a self-developed version of the ADD algorithm for warehouse location problems, as it is present for clustering sensors to the nodes optimally and to optimise the node positions. A configuration consisting of ten strain gauges on three sides of the tube and three sensor nodes on the top surface of the tube is derived. This set-up is then realised for a real-world test on the test bench by Arduino Nano 33 IoT-based sensor nodes and HX711 A/D converters and amplifiers. All ten strain gauges are installed on the part and connected to the other components. As a test, a gradually, stepwise increasing load is then applied on the mounted tube, for which the strains are measured at the sensor positions. These strains are sent as data packages to the Flask server of the developed tool to be visualised on the HTML interface as strain-over-time plots. An additional window with the PyVista plot of the deformed shape is shown as well for the live monitoring of the structural behaviour.

In the discussion, different possible influences causing inaccuracies and failures were mentioned to be considered regarding the results. In future works, these influences should be addressed further. First of all, it is identified that the iPINN has problems dealing with the measurement data containing noise and with oscillating signals due to the test bench control, as well as maybe from the iPINNs not optimally chosen sensor positions. To address this issue, the iPINN will be trained on the generated datasets in future works. Besides, in future works, the step from a DD-iPINN to a data-assisted iPINN (DA-iPINN) will be addressed, as in Wang et al. 2024 [34]. By doing so, physically correct and less dependent predictions should be derived, while the use of the generated data ensures correct predictions in the presence of real-world influences on measurements. Furthermore, the architecture is going to be changed to an encoder-based architecture, which allows for application in an optimal sensor placement (OSP). Therefore, the influence of real-world, noisy measurement data and non-optimal sensor positions should be overcome. Besides, the discussion revealed potential for further proving the methodology’s benefit in sensor node placement optimisation, which reduces measurement noise influence by positioning sensors with lower SNR more effectively. Therefore, future works will consider this influence by investigating the effect on signal accuracy for different distances from the sensor node to the microprocessor. From these tests, it can be investigated whether applying the methodology results in a significant benefit for signal accuracy and for which distances to the sensors it might be relevant to apply and for which not. Another aspect of the discussion of the methodology itself is the evaluation of another, similar approach, including the sensor node placement optimisation directly into the OSP. This idea will be addressed in future works as well by including another fitness function into the optimisation, which evaluates how well sensors are clustered for being positioned close to the microprocessors as nodes. By doing so, sensor configurations are preferred that yield high accuracy results and allow for an easier installation of the WSN, with reduced cable lengths from longer distances between sensors and microprocessors. Therefore, it is also important to include geometric constraints resulting from sensor size, the size of A/D converters with quarter bridge completion, and the microprocessors themselves in the optimisation process to ensure the installation remains possible when sensors are clustered closely. The discussion also revealed potential for improving the entire WSN, particularly in terms of installation effort and component interfaces. Therefore, in future work, self-designed printed circuit boards (PCBs) will be used, for example, to have a single component for the quarter bridge completion and the HX711, reducing noise in the system coming from cable connections, possible cable movements, and variations in the soldering quality. Furthermore, the terminal board for the Arduino Nano 33 IoT with the connector clamps is suitable for the prototype for functional validation but is not suitable for ongoing investigations and tests. Therefore, a new terminal board will be designed, which allows for separate screw terminal blocks for each sensor, allowing better and clearer cable management. Furthermore, it allows to have reduced cables to the ground and a 3.3 V power supply, since these will then be integrated into the PCB itself. Besides, the Arduino Nano 33 IoT should be replaced by a smaller microprocessor, such as the ESP32-C03, since the Arduino has many unnecessary functions and inputs for this application, including those for analogue signals. Therefore, smaller sensor nodes can be realised, which makes the installation easier on parts like the considered aluminium tube. Besides, further investigation of the robustness of these optimised placement solutions will be investigated in future works. For this purpose, the analysis of the shape sensing accuracy in the presence of signal drift or failure of one or more sensors will be investigated to ensure high reliability. Therefore, a combination with the reliability analysis of WSNs as proposed in [41] should be realised to include reliability-oriented OSP into the process. Lastly, the use of the MM and its robustness to errors between the FEM model and the real-world measurements will be investigated in future works. For this purpose, a sensitivity study on the degree of allowable deviation between reference strains during the OSP and in reality measured strains should be carried out. From this study, a further robustness quantification, as in [26], in terms of material characteristic deviations, might enhance the decision-making for the best-suited shape sensing methods for real-world applications.

## Figures and Tables

**Table 1:** Comparison of measured strains of all sensors for a load of 12 mm in contrast to the results from an FEM simulation.

Sensor ID	Measured Strain [µm/m]	Simulated Strain [µm/m]
SG11	117.19895	180.4
SG12	−52.65185	−80.5
SG13	67.4118	102.5
SG14	44.29854	68.3
SG21	152.41414	227.9
SG22	24.37343	37.4
SG23	40.59444	58.2
SG31	4.61618	6.3
SG32	−21.03724	−55.7
SG33	1.03086	−7.5

## Data Availability

The measured data from the test case study as well as the FEM simulation data are openly available at the Research Data Repository of the Leibniz University Hannover at https://doi.org/10.25835/oakhkxtm (accessed on 30 July 2025).

## Abbreviations

The following abbreviations are used in this manuscript:

3D	Three-dimensional
*a_Noise_*	Noise amplitude
ANN	Artificial Neural Network
A/D	Analogue/digital
CAD	Computer-aided design
CFRP	Carbon fiber reinforced plastic
E	Young’s modulus
$E_{r,i}$	Energy contribution of mode *i*
*f*	Objective/fitness function
*F*	Force
FEM	Finite element method
FOS	Fiber optical sensor
GUI	Graphical user interface
HTML	Hypertext markup language
iFEM	Inverse Finite Element Method
iPINN	Physics-informed Neural Network for inverse Problems
IoT	Internet of Things
*L*	Number of layers
L	Loss during training
M	Number of modes
MM	MM
*n*	Maximal number
*n_Gen_*	Number of generations
NSGA-II	Non-dominated Sorting Genetic Algorithm II
OSP	Optimal Sensor Placement
PC	Personal computer
PCB	Printed circuit board
PINN	Physics-informed Neural Network
ReLU	Rectified linear unit function
S	Number of strains
SHM	Structural health monitoring
SNR	Signal-to-noise ratio
q	Modal coordinates
u	Vector of displacements
u,v,w	Displacement components in *x, y, z* direction
UML	Unified Modeling Language
WLAN	Wireless local area network
WSN	Wireless Sensor Network
x	Neural network input
*x, y, z*	Directions in the three-dimensional coordinate space
%ERMS	Percentage root mean square error
ε	Vector of measured strains
$\varepsilon_{xx}$	Strain in *x* direction
ϑ	Activation function
μ	Mean value
ν	Poisson ratio
σ	Standard deviation
$\phi_{d}$	Displacement modal shape matrix
$\phi_{s}$	Strain modal shape matrix
ω	Angular frequency
L	Loss function

## Appendix A

This Appendix shows the values for the SNR considered during the optimal sensor node placement in comparison to the SNR observed during the tests for the max. applied load. For the expected SNR, the calculation is carried out according to Equation (11), and for the real evaluation, in accordance with Equation (17).

(17)
$$SNR\left[dB\right]=20\cdot \log_{10}\left(\frac{\mu_{Measurement}}{\sigma_{Noise}}\right)$$

The different equations are used because the OSP processes utilise a predefined noise amplitude, and the data analysis of the test data was carried out for a series of measurements of time. A comparison of both is shown in Table A1.

From the comparison in the table, it becomes clear that the real SNR is a lot higher than the expected one, which indicates a better noise level than expected. Therefore, the node placement is based on a more conservative estimation, which yields results with higher safety factors.

**Table A1:** Comparison of SNR values between values used during node placement optimization and values resulting during the experiment.

Sensor ID	Expected SNR [dB]	Real SNR [dB]
SG11	35.35787	55.7515179
SG12	28.40767	49.1740088
SG13	30.55412	51.692899
SG14	26.90719	47.5269178
SG21	37.63991	58.9215918
SG22	21.71773	42.4208688
SG23	26.14873	47.2005006
SG31	7.265055	27.8748798
SG32	20.43918	41.5085269
SG33	5.75661	16.6517324
