# Correction: Meyer zu Westerhausen et al. Optimisation of Sensor and Sensor Node Positions for Shape Sensing with a Wireless Sensor Network—A Case Study Using the Modal Method and a Physics-Informed Neural Network. *Sensors* 2025, *25*, 5573

**DOI:** 10.3390/s25216548

**Published:** 2025-10-24

**Authors:** Sören Meyer zu Westerhausen, Imed Hichri, Kevin Herrmann, Roland Lachmayer

**Affiliations:** Institute of Product Development, Leibniz University Hannover, An der Universität 1, 30823 Garbsen, Germany

Mistakes occurred in the original publication [1]. We would like to adjust the discovered mistakes with this correction of the original publication. One mistake refers to Equation (1) of the publication in Section 2.1.1. In this equation, the modal coordinates q were omitted from the equation. Therefore, it has to be corrected to the following Equation (1):

(1) $$u=\;\phi_{d}q$$

Another mistake refers to the results of the application of the modal method (MM) with the measured strain values. This mistake comes from a wrong assignment of the sensors to the corresponding elements in the FEM model. Addressing this issue, a correction factor seems to have been applied in the calculation script. The values for the optimal sensor placement are not influenced. However, this leads to the need for the correction of the following content in Section 4.3 in [1]:

The modal method is influenced by the around 30% to 35% lower strains of the measurements compared to the FEM model as well as the iPINN. However, it still yields more accurate results. For the deformed shape reconstruction, the error in form of the percentage root mean squared error (%ERMS) is *%ERMSx* = 23.66%, *%ERMSy* = 12.21%, and *%ERMSz* = 16.34% for the three displacement components in the directions *x, y,* and *z*. These values are higher compared to the optimal sensor placement (OSP) results, which clearly results from the lower measured strains then in the FEM simulation. These values come from an underestimation of the displacements. This becomes clear from a look at Figure 1, which is an adjusted, corrected version of Figure 13 in [1]. Therefore, it can be observed that the MM shows very good correspondence with the displacement distribution of the FEM reference solution, but clearly underestimates the displacements in comparison to the lower measured strains.

From this comparison, it becomes even more clear that further development of the wireless sensor network is necessary to avoid such errors of displacement field reconstruction.

The authors state that the scientific conclusions are unaffected. This correction was approved by the Academic Editor. The original publication has also been updated.

## Figures and Tables

**Figure 13:**
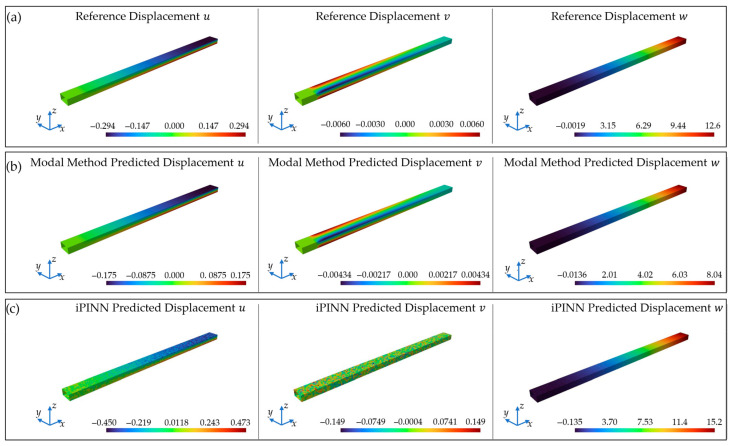
Comparison of the displacement fields for the displacement components *u*, *v*, and *w* for (**a**) the FEM simulation with 12 mm displacement load, (**b**) the MM field reconstruction, and (**c**) the iPINN field reconstruction from the WSN measurements.
